# Going through laparoscopic liver resection in patients with colorectal liver metastases—A qualitative study

**DOI:** 10.1002/nop2.206

**Published:** 2018-11-20

**Authors:** Camilla Bøe, Hilde Bondevik, Astrid Klopstad Wahl, Marit Helen Andersen

**Affiliations:** ^1^ Department of Health Sciences University of Oslo Oslo Norway; ^2^ Division of Surgery, Inflammation Medicine and Transplantation Oslo University Hospital Oslo Norway

**Keywords:** colorectal cancer, interviews, laparoscopic liver resection, patient experiences, qualitative method

## Abstract

**Aim:**

Colorectal cancer is one of the most common cancers worldwide. Surgery is seen as the only curative treatment. There are two approaches to liver resection: open or laparoscopic surgery. Knowledge from the patient perspective can illuminate how it is experienced going through laparoscopic surgery. We aimed to study patient perspectives of the experience of undergoing laparoscopic liver resection surgery in patients with colorectal liver metastases.

**Design:**

This study has a qualitative research design. Nine patients participated in semi‐structured interviews 6 months after surgery. Data were analysed according to Kvale's five‐step analysis method.

**Results:**

Though the patients were satisfied with the laparoscopic approach, they expressed unmet informational needs about the new technique, time after discharge and surgery outcomes related to having metastatic cancer. Healthcare professionals should provide information and support that recognizes the needs of patients with cancer undergoing laparoscopic liver resection surgery.

## 1 INTRODUCTION

Colorectal cancer is one of the most common cancers worldwide (Arnold et al., 2016). Also, in Norway where colorectal cancer is the fourth most common cancer type (Cancer Registry of Norway, 2013) and as much as 30% of patients already have liver metastases by the time, they are diagnosed (Sorbye, Braendengen, & Balteskard, 2008). Surgery is considered to be the only curative treatment with 5‐year survival rates of 40%–57% (Kanas et al., 2012, Tamandl et al., 2007, Van den Eynde & Hendlisz, 2009). There are two approaches to liver resection: laparoscopic and conventional open surgery (Kazaryan, Marangos, et al., 2010). The number of malign tumours and tumour placement in the liver impacts on what surgical method to choose for the individual patient. Hence, the responsible surgeon plays an important role in the decision on surgical approach. Laparoscopic resection for colorectal liver metastases has been an option in Norway since 1998, and an increasing number of patients with liver metastases after colorectal cancer is treated using the laparoscopic technique (Kazaryan, Rosok, & Edwin, 2010). Previous research has shown the laparoscopic approach to be comparable to open surgery concerning oncological results (Hasegawa et al., 2015; Lewin et al., 2016). Several benefits are associated with the laparoscopic method compared with open surgery: less post‐operative pain, faster recovery and earlier return to work and activities of daily life (Mirnezami et al., 2011). Research has also found that hospitalization is shorter after laparoscopic liver resection, compared with open surgery (Vanounou et al., 2011). When it comes to costs, the conventional open approach seems to be the best alternative. However, if the costs of a longer hospitalization are accounted for, the laparoscopic liver resection technique is presumed to be more cost‐effective (Nguyen, Gamblin, & Geller, 2009).

Previous patient perspective studies in the context of colorectal liver metastases have focused on the experiences of being diagnosed with cancer and undergoing surgery (Mizuno, Kakuta, Ono, & Inoue, 2007), as well as patient experiences of going through liver transplantation as a treatment option (Vidnes, Wahl, & Andersen, 2012). Mizuno et al performed a qualitative interview study to understand the experiences of patients going through surgical resection of colorectal cancer. The researchers in‐depth interviewed seven male and six female patients finding that patients felt vulnerable and reported lack of control during the 6 months after cancer surgery (Mizuno et al., 2007). In the study of Vidnes et al., aiming to explore patient experiences following liver transplantation due to liver metastases from colorectal cancer, nine in‐depth interviews were carried out. Overall, the patients, being interviewed 6 months after surgery, expressed that having undergone liver transplantation was a positive experience related to experimental treatment that might prolong life (Vidnes et al., 2012). Very few studies considering laparoscopic liver resection from the perspectives of patients undergoing this new technique have been identified. However, we found a study of Vandrevala, Senior, Spring, Kelliher, and Jones (2016) aiming to ascertain patient experiences of early discharge following enhanced recovery programme for liver resection surgery. Twenty patients were interviewed preoperative, then 6 weeks’ post‐surgery. Though many of the patients felt positive about having an early discharge, some concerns about being discharged early were reported, like worries about coping outside the hospital and concerns about readmittance. Hughes, Knibb, and Allan (2010) reported on a phenomenological study focusing on patient experiences of women undergoing laparoscopic surgery for endometrial cancer (Hughes et al., 2010). Results from the 14 in‐depth interviews indicated that the presence of cancer, fear and unmet informational needs overshadowed the advantages of the laparoscopic approach (Hughes et al., 2010).

In the research literature, there is a lack of knowledge illuminating the patient perspectives of going through laparoscopic liver resection. In general, stories from patients provide important personal and contextual knowledge of human experiences (Tong, Chapman, Israni, Gordon, & Craig, 2013). When it comes to introducing new surgical techniques, the use of patient preferences is crucial to provide good health. The knowledge gathered from patient experiences of their situation, the treatment they go through and the different phases of patient pathways is important in building good quality healthcare services. Also, such knowledge can guide health professionals in how to provide understanding and support. To fully understand the impact of introducing laparoscopic liver resection in patients with colorectal liver metastases, we need better understanding of the complexities of undergoing this new surgical technique as seen from the patients’ perspective. Hence, we aimed to study patient perspectives of the experience of undergoing laparoscopic liver resection surgery in patients with colorectal liver metastases.

## 2 METHODS

### 2.1 Design

This study employs a qualitative approach to study patient perspectives of the experience of undergoing laparoscopic liver resection surgery in patients with colorectal liver metastases.

Thus, a design based on semi‐structured interviews was used to identify the participants’ experiences and their specific perspectives as they explain and talk about them. In line with this study's methodological framework and research questions, we adopted a phenomenological experience‐based approach to illness and health in accordance with Kvale and Brinkmann's (2009) and Malterud's (2012) understanding and recommendation of it. This means that the research aims to investigate individual and unique human experiences (phenomena) as these manifest themselves in daily life and specific situations. In our study, this entails an exploration of the experience of undergoing laparoscopic liver resection from a patient perspective.

### 2.2 Participants and setting

All patients eligible for laparoscopic surgery were informed by the responsible surgeon concerning the new technique, including possible risks, benefits and drawbacks. On this background, the decision of surgical approach was made between the patients and the surgeon.

The participants were recruited from one hospital in Norway, and the inclusion criteria were as follows: (a) patients with detected liver metastases after colorectal cancer; (b) patients who had undergone laparoscopic liver resection at one hospital in Norway during 2011 being 6 months from surgery; (c) patients aged 18 or older; and (d) patients are able to understand and speak Norwegian. Fourteen patients fitting the inclusion criteria in the selected period were invited to participate. All fourteen were sent a letter including information about the study, a reply slip and a prepaid response envelope. Nine out of 14 patients agreed to participate. There were different reasons for patients refusing to take part of the study: living far away from where the interviews took place, not willing to be interviewed by phone and feeling too old or weak to participate. One patient refused without giving a reason.

One of the researchers (CB) contacted the participants by phone on receiving the reply slip. During the phone call, arrangements for the interview were made and the participant was given a chance to ask questions about the study. Some had undergone major surgery between the laparoscopic liver resection and the time of the interview. All together, every participant had various experiences of living with cancer over time, the impact of metastatic disease and different kinds of cancer treatment. Participant characteristics are described in Table 1.

**Table 1:** Demographic variables (*N* = 9)

Variables	*N*
Age	
25–35	1
36–45	0
46–55	0
56–65	4
66–75	2
76–85	2
Gender	
Male	5
Settlement	
Northern Norway	1
Southern Norway	0
Eastern Norway	7
Western Norway	0
Central Norway	1
Diagnosis primary tumor	
Colon	4
Rectal	5
Surgical technique‐primary tumour	
Open	3
Laparoscopic	6

The day before surgery, the patients were informed about the surgery and the post‐operative pathway. This included information concerning the surgical technique, possible risks and complications per‐ and post‐operatively, post‐operative patient care including pain management and a plan for discharge. The patients received preoperative premedication, anaesthesia and post‐operative pain management guided by a standardized protocol. Post‐operative pain regime at day 1 (surgery day) was paracet 1 g × 4 and toradol 30 mg × 3 and from rom Day 2 toradol 30 mg × 3. All patients had urine catheter established during surgery, being removed within three hours after surgery. No drain was established. The patients normally had their own clothes on during the evening of day 1. Criteria for discharge were eating and drinking normally, performing activity of daily living, being able to walk alone and no nausea. Usually discharge day was at Day 2 (the day after surgery).

### 2.3 Interviews

The interviews were performed during 2011 using a semi‐structured interview guide, based on the researchers’ clinical experiences of caring for patients going through laparoscopic surgery, as well as review studies of outcomes after laparoscopic and open liver resection (Mirnezami et al., 2011; Vanounou et al, 2011). The interview guide covered themes related to physical, mental and psychosocial issues (Table 2).

**Table 2:** Interview guide

	Topics
Introduction	Living situation, family, job status, diagnosis and treatment
Physical function	Physical function: pre‐ and post‐operatively, during rehabilitation
	Post‐operative pain, physical symptoms
	Physical impact of surgery on activities of daily living, and job situation
Mental function	Coping with cancer diagnosis, treatment and prognosis
	Thoughts and emotions related to going through laparoscopic versus open surgery
Social function	The impact of laparoscopic surgery on social life during rehabilitation period
Information and care provision	Informational needs before and after surgery, at hospital discharge, and during rehabilitation. Experiences with information and care delivered by health care professionals

Two researchers (CB, MHA) conducted the interviews approximately 6 months after surgery. Eight interviews took place in a closed room with a relaxed atmosphere at the hospital where the surgery was performed, whereas one interview was performed in the home of the participant in accordance with the participant's own will. A time frame of 6 months after surgery was considered as the best time to interview, because participants had had the time to recover mentally and physically, but still remembered their experiences from the time before and after surgery. All interviews were audiotaped and lasted approximately 30–70 min. The interviews, which were planned, yet flexible, aimed to collect the participants’ descriptions and perspectives of how they experienced the laparoscopic liver resection (Kvale & Brinkmann, 2009). All interviews began with a short briefing about the purpose of the study. During the interview, the researcher asked follow‐up questions, so the informants had to further elaborate on the statements they had made. Probes were used to stimulate narration, such as “What did you think then?” After the interview, participants were given the opportunity to ask questions and seek clarification if they felt anything was left unclear.

### 2.4 Ethical considerations

The study complied with the guidelines of the Helsinki convention throughout the entire research process (World Medical Association, 1983). Approval was obtained from The Institutional Review Board at Oslo University Hospital, number #2011/14005.

### 2.5 Data analysis

The first author transcribed all interviews verbatim. The interviews were then analysed by CB and MHA following an inductive, thematic strategy and using Kvale's 5 steps for meaning condensation and interpretation in qualitative data (Kvale & Brinkmann, 2009). Step 1 was to read all the transcribed text to get an overall impression. During step 2, the transcribed text was perused in more detail, looking for meanings in the interviews. The text gave an impression of what the participant actually meant by saying what he/she did. During step 3, we formulated and categorized the themes dominating a meaningful unit and tried to state these as simply as possible. The aim of step 4 was to consider the main themes and see if they corresponded with the purpose of the study. Hence, this step consisted of interrogating the meaning units in terms of the specific purpose. The themes of the meaning units were addressed with respect to questions such as, “What does this statement tell about the patients’ perspectives of the experience of undergoing laparoscopic liver resection surgery?” Step 5 aimed to tie the major themes into a descriptive statement. Quotes derived from the interviews were used to illustrate the themes (Kvale & Brinkmann, 2009). Example from the data analysis process is shown in Table 3.

**Table 3:** Example from the data analysis process

Natural meaning units	Subthemes	Theme
“I underwent surgery early in the morning and I was back in my own clothes nearly immediately afterwards, sitting in a chair in my room at surgical unit. So a keyhole operation was a positive experience.” (I: 04) “It went quite quickly, and I remember that my family was surprised about this. I did not stay very long in the recovery unit, and was transferred back to the surgical unit shortly afterwards. I sent messages by my mobile phone telling my family that everything had gone well. They were surprised about how fast I recovered, and that I was able to send messages within a couple of hours after the operation.” (I: 01) “So I cannot remember that I had any pain. No, I didn't have any pain. I remember I was asked at the hospital if I had any pain in my shoulder. I once experienced having shoulder pain during one of my laparoscopic operations, but cannot remember which of the operations it was. When it comes to the incisions, I did not experience any pain at all” (I: 01) “Yes, it was painful, but I had pain killers to remove the pain. The pain was worst in the navel area. However, I had been through abdominal surgery 6 weeks before, therefore getting in and out of bed was painful. However, previously I had a herniated disc, and the surgical pain following laparoscopic method was nothing compared to that.” (I: 03)	Experiencing a rapid recovery Experiencing minor pain easy to cope with	A rapid recovery with minor pain

### 2.6 Trustworthiness

We used different strategies to help ensure rigour throughout the study. When working out the research question and the interview guide also clinical experts were contacted to get peer opinions. These were all experienced nurses or surgeons affiliated at the department where the informants had undergone laparoscopic liver resection surgery. The researchers performing the interviews had different tasks, one of them interviewing (CB) and the other insuring integrity of the participants and adding supplementary questions when necessary (MHA). In the first phase of the data analysis, two members of the research group knowing the field especially well (CB, MHA) met regularly to discuss the data and make categories and propose preliminary themes. This implied switching back and forth between the transcripts and themes to assure an accurate reflection of the interview data. Then, a third researcher not affiliated to the clinic (AKW) joined the group and questioned the preliminary analysis. The three researchers then discussed carefully alternative ways of interpreting, categorizing and organizing the data until consensus was reached.

## 3 RESULTS

Three main themes were generated through the analysis process. The first theme, “A rapid recovery with minor pain” described experiences during the first post‐operative days and how the participants experienced to recover from surgery. The second one, “Beneficial recovery versus uncertainty of a new technique for cancer treatment,” deals with how most of the participants were satisfied with a rapid recovery but at the same time experienced worries whether the new surgical technique would affect their cancer negatively. The third theme, “Unmet informational needs during hospitalization and after hospital discharge” described the participants’ experiences of not getting sufficient information from health personnel during hospitalization and at discharge.

### 3.1 A rapid recovery with minor pain

All of the participants expressed that they were back to their normal routines quite quickly after surgery and that the need of hospitalization did not last long. The participants returned to the surgical unit only a few hours after surgery and were back on their feet after only a short period of time. The need of physical nursing care was minimal, and the participants were in their private clothes within a short period after surgery. Getting quickly back to normality was considered favourable, as one of the informants in the study articulated:

I underwent surgery early in the morning and I was back in my own clothes nearly immediately afterwards, sitting in a chair in my room at surgical unit. So,, a keyhole operation was a positive experience. (I: 04)

And another one:

It went quite quickly and I remember that my family was surprised about this. I did not stay very long in the recovery unit and was transferred back to the surgical unit shortly afterwards. I sent messages by my mobile phone telling my family that everything had gone well. They were surprised about how fast I recovered and that I was able to send messages within a couple of hours after the operation. (I: 01)

Through the interviews, it became clear that the participants quickly got back to normal routines also when it came to eating and drinking. Most of them stayed in the hospital only for a day or two after surgery and were directly discharged to their homes. The participants talked about the first 2 weeks after surgery; some went back to work only a week post‐operatively, whereas others needed more time at home. One participant described the first period after discharge: “After a week I was back to normal, working full‐time.” (I: 05).

However, it also derived from the interviews that the time after discharge could be challenging:

On Monday I underwent surgery and on Tuesday I was discharged from hospital. That wasn't a good experience. The following Monday I took my first walk and after a fortnight I could take walks as usual. (I: 06)

In general, the study showed that most of the participants left the hospital within 2 days following laparoscopic liver resection. Within 2 weeks, most experienced to be back to daily life. Although some felt the first few days at home were tough, in retrospect, this was an easy period compared with previous cancer treatment they had endured.

The participants generally experienced little post‐operative pain, varying from no pain at all, to some pain from the incisions. Few months after surgery, pain was the topic the participants struggled the most to remember:

So, I cannot remember that I had any pain. No, I didn't have any pain. I remember I was asked at the hospital if I had any pain in my shoulder. I once experienced having shoulder pain during one of my laparoscopic operations but cannot remember which of the operations it was. When it comes to the incisions, I did not experience any pain at all. (I: 01)

For some participants, it was hard to know if the pain they felt was caused by previous surgical procedures. Retrospectively and considering previous experiences from laparoscopic surgery, they described these as surgical interventions without huge pain. One expressed it like this:

Yes, it was painful, but I had pain killers to remove the pain. The pain was worst in the navel area. However, I had been through abdominal surgery six weeks before, therefore getting in and out of bed was painful. However, previously I had a herniated disc and the surgical pain following laparoscopic method was nothing compared to that. (I: 03)

None of the participants mentioned that they had abdominal pain that lasted for months after the laparoscopic liver resection or were in pain at the time of the interview.

### 3.2 Beneficial recovery versus uncertainty of a new technique for cancer treatment

To be operated by laparoscopic technology was seen as beneficial compared with the conventional open surgery when it came to a convalescence and pain:

About the laparoscopic surgery, it was a better experience than I expected. Now, I see it as a benefit that the surgeons are able to use this technique. I don't know much about how it happens, but it was a great advantage. (I: 01)

Thinking of the surgeons carrying out the operation using keyhole technology, I feel I have won the lottery, so I am very pleased. (I: 09)

However, for some participants, the joy of a rapid recovery was overshadowed by uncertainties and dissatisfaction with laparoscopy as a method for cancer treatment. Worries and negative thoughts were expressed about the oncological aspect of the surgery:

Yes, it was an OK operation, where recovery was quite quick. However, when I went through major surgery some months earlier, the surgeon saw two lumps on the liver. One of them they were sure to be cancerous, but they were not sure about the other one. I therefore had keyhole surgery and then one month ago when they operated the other part of the liver, they found several lumps in it. I would had preferred it if they had opened me completely and checked me thoroughly instead of using a keyhole surgery inspection. So afterwards, I feel that they did an easy operation, which they weren't sure about, even though recovery was quicker after the keyhole surgery. If it is done correctly, the keyhole surgery is an advantage. But I also think that it is an easy way out for the hospital. (I: 07)

However, I must admit that I was a bit uncertain when they informed me about removing a small lump on my liver using the keyhole technique. I was skeptical, thinking it was not safe for me only removing the lump. After this operation I often think that the cancer will come back. For the future, I would prefer to have open surgery. (I: 08)

### 3.3 Unmet informational needs during hospitalization and after hospital discharge

The information the participants received was often highlighted and a huge topic in the patient stories, about which everyone had a lot to say. There were references to disappointment and displeasure related to the lack of information. During the interview, we talked about the information given before the operation in preparation for the surgery itself. We also talked about information and instructions during hospitalization. The information given about potential outcomes, further progress and discharge from the hospital was also natural topics. The participants indicated that the information they received before surgery was sufficient, but they received very little information about how to cope after discharge which in turn made them feel uncertain:

I can say that I received sufficient information before surgery. I did not receive much information about what would happen after surgery. There perhaps isn't much to say, but how should you behave after such an operation? Can you work out at a gym? Should you be as active as possible? Are there types of food you should avoid when you have had bowel problems? I asked about such things and I should have received something in writing about what to do. It is possible that you should try to live as normally as possible, but they could have told me. I also don't think that I have received any information about what to expect for the future. (I: 04)

Lack of information about their own situation and the challenges in daily life seemed to worry most of the participants. They constantly had to keep track of when they had appointments, and some had to ask to get the results of the operation:

There has been an unbelievable lack of information. You need to be healthy and strong in order to tolerate this. You become very insecure because of this [lack of information]. Our family has been able to cope with this, but it hasn't been easy for us either. I have tolerated all the operations, but that is not the problem. Not knowing, not hearing is not all right. This wears me down and so does it for those around me. (I: 03)

Right after surgery, the surgeon talked to the patient about the operation and how it went. Information about the removed tumour was also given. However, when it came to the specific findings about the type of tumour cell, potential results from surgery and information available some weeks post‐operatively, the participants expressed unmet informational needs:

I had to call the hospital to get an answer concerning malignancy of the tumor. Then they told me that the tumor was malignant. They told me that they thought I already knew, because they had removed it so quickly. However, they apologized slightly that I had not been told. (I: 09)

## 4 DISCUSSION

The findings from this study showed that patients with colorectal liver metastases underwent an operation described as beneficial concerning recovery. According to them, this was a major benefit of laparoscopic surgery. It was of great importance to recover quickly and to return to normal daily lives as soon as possible. The benefits to laparoscopic surgery are confirmed in previous studies. A quantitative study carried out on laparoscopic liver resection demonstrated that this surgical approach entailed fewer complications and less pain (Kazaryan, Marangos, et al., 2010). A comparative study of patients going through open and laparoscopic colorectal resection documented less need of physical care from the healthcare providers in the laparoscopic group (Richardson & Whiteley, 2011). This study's findings showed that the patients’ physical care needs were minimal following this type of surgery. Kehlet and Wilmore (2008) confirmed that after laparoscopic liver resection, it is feasible to discharge the patient from the hospital within 2 days.

Living with cancer itself is probably a significant psychosocial and existential burden and may indicate the importance of being able to live one's life as normal as possible. Returning to work was a topic that often was brought up during the interviews in our study. A study done on colorectal cancer survivors showed that as many as 89% returned to work after their cancer diagnosis. Returning to work was important for the patients’ well‐being (Sanchez, Richardson, & Mason, 2004).

Post‐operative pain has always been a major concern for patients, and in this study, the majority experienced minor post‐operative wound pain. Our findings support the results reported by Hughes et al., (2010) as the participants in our study concluded laparoscopy involved less pain compared with other surgical techniques and pain experiences. In a study of Andersen et al. (2006), a total of 122 patients were randomized to open or laparoscopic donor nephrectomy. The results showed fewer morphine equivalents admitted to the laparoscopic group on the day of surgery. One month after surgery, the patients in the laparoscopic group reported less pain compared with the patients in the open group. None of our 9 participants described long‐lasting pain 6 months after surgery. This is in line with a study documenting that about 10% of patients would develop long‐lasting pain after surgery (Carroll et al., 2013).

The uncertainty concerning laparoscopy as a method was something that became apparent in the interviews. This has also been documented in earlier research. The study of Hughes et al. (2010) also documented that the experience of undergoing laparoscopic surgery was overshadowed by the fear of cancer and unmet informational needs. Concerns about curative uncertainties were also reported in a study of patients undergoing conventional open liver resection (McCahill & Hamel‐Bisell, 2009). The study showed that patients with cancer experience substantial psychological stress related to the disease itself, future prospects, the treatment and its side effects. During the last decade, laparoscopic liver resection has become a routine procedure at many hospitals worldwide. Our study represents the period when this method was introduced, and the findings related to uncertainty concerning laparoscopy as a method must be interpreted in that context. As laparoscopic liver resection now is considered a standard surgical procedure probably, a similar study of today would reveal less concern concerning uncertainty of the laparoscopic technique.

Lack of information about their own situation and the challenges in daily life seemed to worry most of the participants. Our study illuminates the need of thorough information and continuity of care also after hospital discharge.

### 4.1 Study limitations

A limitation of our study is the small sample size. This study was based on nine patients going through laparoscopic liver resection. Although we initially planned for a larger sample size, we argue that data from the nine participants provided us with relevant, new knowledge and deeper insight into the situation of undergoing laparoscopic liver resection surgery. The patients represented a broad variation in demographic characteristics, such as age, gender and settlement (Table 1), also in line with the variations in this patient population in general. By reflecting characteristics of the typical laparoscopic liver resection patients, this study made a positive contribution to existing knowledge in this area. However, it is possible that more informants would provide multiple nuances and variations to the results. Hence, our findings should not be generalized. Nevertheless, this study has shed light on the meanings expressed by patients undergoing laparoscopic liver resection. As such, our findings represent new, relevant knowledge into the situation of undergoing laparoscopic liver resection, relevant both for potential patients and health professionals.

One of the researchers (CB) was partly in charge of the care of one study participant. To balance dual roles as both a clinician and a researcher can be a dilemma. While interviewing, it may be difficult to maintain the role as a researcher. To reduce possible biases, two researchers performed the interviews, the second researcher not being in charge of care.

### 4.2 Recommendation for practice

This study adds new important information to the existing knowledge about laparoscopic liver surgery as seen from the patients’ own perspective. Our data support the notion that the laparoscopic approach is an attractive alternative to open liver resection due to minor pain and a rapid recovery. Thus, it is important to expand this procedure. However, it should be kept in mind that this method is still evolving. Long‐lasting experience in the field and technical experience is needed for a successful laparoscopic approach.

The findings of our study demonstrate how important it is to provide patients with detailed information about the disease, the type of treatment they receive and to follow them up during their stay at the hospital and after discharge. When new surgical techniques are introduced, it is crucial to meet the patients’ informational needs. Healthcare providers must not make assumptions or take anything for granted, and this is a significant challenge that needs to be addressed. The understanding of the uncertainty associated with the method is little explored, and the area warrants further research.

Data from the semi‐structured interviews support providing balanced patient information about the risks, drawbacks and benefits. According to the results, information should be provided both before the hospital stay, pre‐ and post‐operatively and after discharge.

Finally, having metastatic cancer and undergo a new surgical method may increase the patient's distress. Thorough information from an oncologic perspective, existential support and the continuity of care after hospital discharge are important.

## 5 CONCLUSION

Though the patients were satisfied with the laparoscopic approach, they expressed unmet informational needs about the new technique, time after discharge and future prospects related to having metastatic cancer. Healthcare professionals should provide information and support that recognizes the needs of patients with cancer undergoing laparoscopic liver resection surgery.

## CONFLICT OF INTEREST

None declared.

## AUTHOR CONTRIBUTIONS

CB, MHA and AKW: Responsible for the study conception and design. CB and MHA: Responsible for data collection. CB, MHA and AKW: Responsible for the data analysis and the manuscript. CB, MHA, AKW and HB: Critical revisions to the paper for important intellectual content.
